# BOUNDARIES, STRUCTURE, AND TURNOVER: A COMPARATIVE ANALYSIS OF OLDER ADULTS’ CORE DISCUSSION NETWORKS

**DOI:** 10.1093/geroni/igae098.1530

**Published:** 2024-12-31

**Authors:** Nell Compernolle, Alyssa Goldman

**Affiliations:** NORC at the University of Chicago, Chicago, Illinois, United States; Boston College, Chestnut Hill, Massachusetts, United States

## Abstract

Decades of research have collected information about individuals’ core discussion networks as a fundamental measure of social integration, with implications for health and well-being. Growing interest in personal network dynamics—additions or losses of key social ties overtime—has sparked recent critiques of the validity and reliability of personal network measurement. Specifically: whether typically-used name generators accurately capture social ties on which individuals ultimately rely in times of need. Here, we compare findings from two longitudinal studies of older adults’ personal networks: the National Social Life, Health, and Aging Project (NSHAP; four rounds of data collection spaced five years apart) and the Chicago Health and Aging in Real-Time (CHART; three waves spaced six months apart). Results suggest that oft-cited concerns of egocentric data collection may be less problematic than recently suggested. Regarding boundary specification, older adults in both studies report an average of 3-4 close confidants, with more than half reporting more than five and, among CHART respondents who do, an average of 1.4 additional confidants. Regarding network structure, the two studies yield near identical results in the proportion of core confidants being kin (~70%), co-residing (~20%), average frequency of interaction (a few times a week), and average closeness (very close). Last, regarding network turnover, we observe slightly fewer additions and losses between waves in the CHART study compared to NSHAP, although both show relatively little turnover. Together, findings support the utility of core discussion networks in capturing older adults’ core confidants at a given point in time.

